# American Academy of Dental Science

**Published:** 1890-02

**Authors:** 


### AMERICAN ACADEMY OF DENTAL SCIENCE.

The American Academy of Dental Science held its regular monthly meeting December 4, 1889, in the Boston Medical Library Association rooms, President Seabury in the chair.

*President Seabury.*—Gentlemen, I will now introduce to you Dr. D. W. Fellows, of Portland, Me., who will read a paper on "Fracture of the Jaws."

*Dr. Fellows.*—I feel considerably abashed before a body of men like this; but such as I have I will give you very briefly. (The paper will be found on page 77).

There are one or two points which I have not touched upon in this paper. One is, the time for inserting the splint. I should say, as soon as possible, but in many cases the swelling would be so great that it would not be well to attempt it for a few days. In the cases I referred to it was inserted in about a week; I think six days. It may be done any time during the preparatory stages, any time within a week, if the circumstances are such that it cannot well be done before, or, in fact, any time before union takes place.

### DISCUSSION OF DR. D. W. FELLOWS'S PAPER ON "FRACTURE OF THE JAWS."

*Dr. Fillebrown.*—I suggest that matters of detail are oftentimes quite as interesting as principles, and I think that the detail of making the splint would be interesting. Not long since I saw a splint that was made for a patient with a fractured under jaw, and the splint was so large that it allowed nearly the full play of the displacement, so that there was but little gained over the condition which would have existed had there been no splint at all. I inquired into the matter, and I found that tin foil, tolerably thick, was moulded over the teeth of the models and wax added to that for the thickness. Then the models were removed from the pattern and the pattern inserted into the flask, the plaster poured into that, and the splint made from the pattern instead of from the model itself. In consequence of the ill-adjustment of the tin foil in making the pattern the splint was loose, as I remarked, and the displacement of the parts allowed. Now, if I understand Dr. Fellows, he used the models themselves on which to make the splint, and by that means he got a more perfect fit. It has always seemed to me in making splints that it might be reasonable to put a coating of thin tin foil over the teeth of the model, and thereby insure clean work, just about enough enlargement to allow it to go easily on the teeth, and still fit closely enough to prevent displacement of the parts. I would not have thought this worth speaking of had I not seen within the year past the very condition which I have alluded to, and I know the patient was in that case very much worse off for the splint's having been made in that way.

The first case Dr. Fellows reports here to-night, that of the merchant of Portland, I was quite well acquainted with. I did not see the case at the time of the accident, but I have seen the gentleman since, and certainly it was a bad smash-up. He fell a considerable distance,—I understood it was from the steps of the cars, but it seems I am mistaken,—and, striking with full force on the face, both jaws were badly fractured. After the treatment no one would know that so severe an accident had happened to any parts about the face.

*Dr. Andrews.*—I have had a case that the paper brings to my mind. It was that of a gentleman connected with Harvard University, who, in trying to stop a vicious, runaway horse, received a kick, which broke his lower jaw, about an inch to the left of the symphysis. Besides the fracture there was a loose, V-shaped piece of the jaw containing three teeth, the cuspid and two bicuspids, I think. A few days after the accident the patient was brought to me by his physician to have a splint made to keep the jaw and teeth in place. The patient having a close-cut beard, we thought it best to cover this with a piece of damp cotton cloth, which was tied round the head like a bandage, one object being to keep the jaws together, and make as perfect an articulation with the upper teeth as possible; another, to prevent the modelling composition from clinging to the beard. A piece of sheet tin, such as we make air-chambers of, was then rudely fashioned to the form of the lower jaw, after which modelling composition was heated, placed in this tin form, carried up in place, and allowed to cool about the jaw. Another bandage was then tied outside of this and over the head. We found that the patient could open the mouth to allow an impression to be made of the teeth, after we had tied the loosened teeth to the firm ones. From the impression a splint of vulcanized rubber was made covering the teeth. In three weeks the splint was removed and the jaw found to be united, the articulation being nearly perfect.

*Dr. Williams.*—I would like to ask Dr. Andrews if he would consider the modelling composition preferable to plaster of Paris?

*Dr. Andrews.*—In this case it worked quite as well, and was found to be much more convenient. I would say that the plate held the jaw so well in place that the outer splint of impression material was soon removed. The only thing being used on the outside was a simple bandage.

*Dr. Smith.*—I was called in consultation with a surgeon some time ago to assist him in holding a double fracture of the lower jaw that the surgeons of the Massachusetts General Hospital had treated unsuccessfully with their usual process of wiring teeth together. The case was that of a gentleman who had been thrown from a carriage, and, like the person whom Dr. Fellows spoke of, he was badly smashed up. Not only was the jaw fractured at the right and left of the symphysis, but the soft parts of the face, especially about the neck, were severely lacerated. The usual procedure of using the interdental splint with a bandage passing under the chin and over the head was prohibited on account of the severe inflammation of the soft parts, producing two sinuses, in which the surgeon had placed tubes for the discharge of pus. I was not called until late in the case, and I found the parts so swollen that it was impossible to take a full impression at once of the jaw. So I took the impression of the lower jaw in three sections by taking modelling compound and using my forefinger as a cup. From these impressions casts were made and put together. A die was then constructed, and on that was struck up a very stiff gold splint going inside of the teeth and over the crowns, but not over the outside. Through this splint, and corresponding to the spaces between the teeth, holes were made, through which passed platinum wire to serve as ligatures in securely fixing the splint in position. Everything being in readiness, the fractures were adjusted, the splint applied and firmly fastened—by means of the platinum ligature—to the teeth. The patient wore the splint some twelve weeks with perfect comfort, and the results were satisfactory to both surgeon and patient.

*Dr. Baker.*—About two years ago a case of fractured jaw came into my hands. A gentleman from this city was travelling through the West. I do not know the exact details of the injury, but was told that he was riding a bicycle, and fell from it on to the sidewalk. The result was two fractures of the lower jaw, one in the vicinity of the condyle on the left side, the other just back of the mental foramen on the right side. Travelling as he was, he could not, of course, remain in any one place for treatment, and therefore went to several different practitioners for aid. One of the dentists whom he consulted made for him an interdental splint, and it was correctly named, but that was all that there was correct about it.

When he came to me, he said he thought he had not been properly treated, and also said, when he reached Chicago, he was in hopes of receiving proper care, and went immediately to Dr. Harland. On learning that he could not remain in Chicago a suitable length of time, Dr. Harland referred him to me. Upon examination, I found a cartilaginous union on the right side. After removing the splint, the articulation was seen to be fairly good on the left side, while on the right there was a space large enough to place a thumb between the upper and lower teeth in the vicinity of the bicuspids. The right inferior second bicuspid and first molar were badly fractured, and the right superior central badly out of position and loose. The pulps of all these teeth were dead, and there was also a discharging fistula under the chin, with considerable necrosis.

The treatment I pursued was as follows: Knowing that several weeks would elapse before the case could be cured, I advised my patient to procure a room at the hospital, and I called to my assistance a celebrated surgeon. We etherized the patient, after which we found that, by exerting considerable force, we could spring the jaw into its proper position.

After removing the necrosed bone, we placed upon the chin a soft pad of cotton, then over this a plaster splint, and finally passed a linen bandage over the head and under the chin, covering the plaster, and drew it tight enough to hold the jaw in its normal position.

The patient was easily fed through the space made by the fracture of the bicuspid and molar. After about three weeks the bandages were removed, and the jaw was found to keep its position so well that it was unnecessary to use the metal shield which had been prepared. The patient merely wore a simple bandage for a few weeks. I then put the central in place, crowned the lower bicuspid, having previously treated and filled the roots. The last time I saw the patient the adhesion was as good as any one could wish, and there was nothing visible to indicate that he had ever received an injury.

*Dr. Pond.*—I had a case, a few years ago, which differed from those already mentioned, in that there were no back teeth on either jaw. The six front teeth were in place in the lower jaw, and it fractured on each side just back of the cuspids, the anterior portion of the bone with the teeth being freely movable.

Bandaging proved useless, for, when the front teeth were brought together, the slightest pressure forced the parts out of place; there being nothing to keep the body of the jaw down, it would be lifted upward into the mouth. An impression was obtained by filling the mouth nearly full of soft modelling composition, holding the parts in position with the hands under the jaw, and gently lifting up until the lower front teeth just articulated with the upper ones.

A splint was made of vulcanite, which resembled a partial upper and under plate joined together,—that is, it covered the upper jaw from the cuspids back to the condyles and the corresponding portion of the lower jaw. Sufficient material was cut away to allow for the passage of food. It was placed in the mouth and the parts firmly bandaged, the splint holding the body of the lower jaw in position, and the front teeth performing the same office for the anterior portion.

The parts were held in position perfectly for about a week, when the patient, an ignorant policeman, who was confined to a hospital bed by some severe bruises received in the same row in which his jaw was broken, decided that the jaw was all right, and removed the bandage and splint. The removal was not discovered until the next day, and as the parts were still in position the splint was not replaced. The liquid diet was continued, and when discharged from the hospital, where his other injuries kept him some weeks, his jaw seemed in as good condition as ever.

